# Membranous Nephropathy: Antigenic Landscape and a Novel Pathogenetic Model

**DOI:** 10.3390/ijms27052423

**Published:** 2026-03-06

**Authors:** Irina Zdravkova, Eduard Tilkiyan, Desislava Bozhkova, Yovko Ronchev, Boris Kirilov, Teodor Kuskunov, Atanas Lambrev

**Affiliations:** 1 Department of Propaedeutics of Internal Diseases, Medical Faculty, Medical University of Plovdiv, 4000 Plovdiv, Bulgaria; 2 Nephrology Clinic, University Hospital “Kaspela”, 4000 Plovdiv, Bulgaria; eet64@yahoo.com (E.T.);; 3 Second Department of Internal Diseases, Section “Nephrology”, Medical Faculty, Medical University of Plovdiv, 4000 Plovdiv, Bulgaria; 4 Department of General and Clinical Pathology, Faculty of Medicine, Medical University of Plovdiv, 4000 Plovdiv, Bulgaria; 5 Department of General and Clinical Pathology, University Hospital “Sveti Georgi”, 4000 Plovdiv, Bulgaria; 6 Clinical Laboratory, University Hospital “Kaspela”, 4000 Plovdiv, Bulgaria

**Keywords:** target antigens, complement pathway, lectin pathway, primary and secondary membranous nephropathy, pathogenesis

## Abstract

Membranous nephropathy is a disease that has been well documented, yet its etiopathogenesis has not been fully clarified and the distinction between its primary and secondary forms has not been completely categorized. The discovery of new antigens and antibodies reveals different percentages of positivity in secondary membranous nephropathy, which is a cause of great confusion and ambiguity not only in diagnosis but also in the choice of a therapeutic approach. The aim of this review is to summarize the literature on newly discovered antigens and antibodies, and to propose a pathogenetic model based on the role of the complement system and its activation pathways. In this model, antigens are categorized based on the type of immunoglobulin deposits and the putative complement pathways that they activate, which can help to differentiate primary from secondary membranous nephropathy. The model also reflects how the deposition of foreign antigens in the basement membrane can activate both the lectin and classical complement pathways, which may explain why positive antibodies are observed in both primary and secondary forms of membranous nephropathy.

## 1. Introduction

Membranous glomerulonephritis has a complex immune pathogenesis, characterized by diffuse subepithelial deposition of IgG and complement fractions. It has been observed that around 75–80% of patients with primary MN (pMN) have circulating antibodies against the phospholipase A2 receptor (PLA2R), while the remaining cases of pMN can be classified as idiopathic MN (iMN) [1]. Secondary MN (sMN) accounts for 20–25% of MN cases and is caused by infections, drugs, cancer, or autoimmune diseases such as systemic lupus erythematosus (SLE), rheumatoid arthritis, urticarial vasculitis, sarcoidosis, thyroiditis, Sjogren syndrome, systemic sclerosis, or ankylosing spondylitis [2]. The incidence of membranous nephropathy in 2011 was 1.2/100,000/year [3], and in 2017, the reported incidence in the United States was 12/million/year, with a median patient age of 50 to 60 and a sex ratio of 2:1, with a male predominance [4]. An increased incidence of MN has been reported in Asia, with air pollution presumed to be the main cause [5,6,7]. A large genome-wide association study discovered an unusual genetic architecture of MN, which explained the increased risk of pMN in East Asians compared to Europeans [8]. These studies prove the importance of genetic predisposition and environmental factors in the occurrence of MN.

MN has been viewed as a condition that predisposes patients towards the development of acute kidney injury (AKI) [9]. There are many mechanisms that contribute towards the potential development of AKI, on the background of MN. The chief and most often observed among them being the prerenal mechanism, caused by low oncotic pressure of the intravascular fluid, which is one of the trademark signs, observed in every nephrotic patient and especially in those with MN, as the condition has the capacity to induce massive proteinuria with a daily loss of more than 20 g of protein. The above mentioned, leads to the transudation of fluid into the interstitium and the depletion of intravascular volume, thus inducing hypoperfusion of the kidneys.

Several other mechanisms have the capacity to contribute towards the development of AKI in MN. The above-described transudation of fluids takes place within renal tissues as well, causing an interstitial edema, which pushes glomeruli further apart from one another (as we can plainly discern, by directly observing fewer malpighian corpuscles in renal biopsy sample cores of nephrotic patients) and more importantly leads to the collapse of renal arterioles, again leading to ischemia. In this case, the induced hypoperfusion (or contribution towards its emergence), should be labeled as a type of renal mechanism.

Another factor, which could facilitate renal AKI in MN is the higher concentration of uric acid. Being a type of chronic kidney disease, MN, leads to the continuous loss of renal function and decline in estimated glomerular filtration rate (eGFR). Around 70% of the urates, which the human body produce, are terminated via the urinary system. The remaining 30% is disposed by being secreted into the gastro-intestinal tract and expelled with the feces. The increase in serum urate concentration is in direct correlation to the decrease in eGFR and has a plethora of effects. First and foremost, uric acid is an intracellular prooxidant, leading to an increase in reactive oxygen species, oxidative stress and cellular damage. The induced oxidative stress causes nitric oxide depletion, which is a key vasodilator in most vessels, including those of the kidney, once again contributing in the disruption of renal perfusion. An additional factor, which also leads to vasoconstriction is that uric acid binds to TLR-4, resulting in inflammasome activation and interleukin 1β production [10,11].

Thromboses of kidney vessels are also viewed as a dramatic cause of renal AKI in membranous nephropathy, being caused by the abnormalities of the coagulation cascade in nephrotic patients. The mentioned vascular incidents can be caused by either an acquired deficiency of anti-coagulant factors (such as proteins S and C), which are lost when a pronounced, non-specific proteinuria is present, or by an excess of produced pro-coagulant factors, observed in patients with specific proteinuria. Their excess being a product of increased synthesis, fueled by the compensatory non-specific anabolic drive in response to the loss of solely low molecular proteins (mainly albumin).

**Figure 1:**
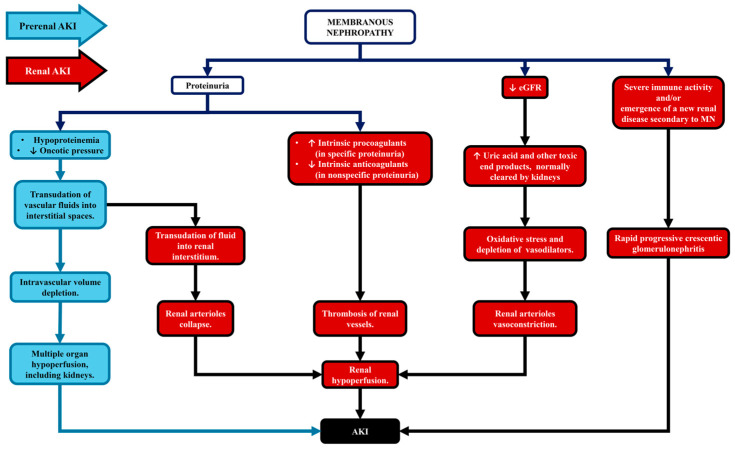
Mechanisms leading to AKI in MN.

Last but not least, while rare, MN may lead to the development of rapid progressive crescentic glomerulonephritis and renal AKI. The above mentioned may come to pass, provided that the immune process is intense enough to rupture the glomerular basal lamina and initiate the generation of crescents. In some cases, anti-glomerular basement disease can be observed as superimposed over MN [12]. The mechanisms leading to AKI in MN are presented in Figure 1.

For over 40 years (between 1959 and 2002), experimental models of Heymann nephritis were considered to most closely and accurately illustrate the pathogenesis of MN in humans. However, despite the extensive search for anti-megalin antibodies in idiopathic MN and attempts to identify megalin as a component of the normal human podocyte cell membrane, no pathogenetic links between Heymann nephritis and human iMN have been established [13]. In early 2002, the research team led by Hanna Debiec and Pierre Ronco published a series of studies that, in many ways, changed our understanding of the pathogenesis of MN [14,15,16]. These authors studied the development of neonatal MN in newborns whose mothers carried a genetic deficiency of neutral endopeptidase (NEP), a membrane-associated podocyte antigen that digests (absorbs) peptides. As the fetus does not lack NEP, feto-maternal alloimmunization occurs and anti-NEP antibodies (often of a very high titer) are produced in the mother. These antibodies (usually of the IgG4 and IgG1 subclasses) cross the placenta and interact with NEP, which is well expressed on the surface of fetal podocytes. Then, in situ immune complexes (containing IgG1 and IgG4) develop in the newborn, resulting in subsequent proteinuria and nephrotic syndromes typical of MN. Of interest is the detection of the C5b-C9 membrane attack complex in the deposits, which suggests that this spontaneous human alloimmune disease may be complement-dependent. The studies by Debiec and Ronco et al. [14,15,16] point to a common feature of MN, which is also a denominator clearly formulated by Kerjaschki [17]: podocytes and their membrane-associated proteins play a central role in the development of the disease by providing antigenic targets for circulating antibodies and for the in situ formation of immune deposits.

## 2. Antigens Associated with MN

The understanding of iMN in humans has evolved from an immune-complex-mediated disease to a podocyte pathology, leading to a search for podocyte-associated antigens for a complete understanding of the disease. This search bore fruit in 2009 with the publication by Beck et al. at the laboratory of David Salant [18], who identified the role of the anti-M-type phospholipase A2-receptor antibody (APLA2R; usually of the IgG4 subclass) in the pathogenesis of iMN in a significant proportion (≥70%–80%) of patients. PLA2R is undoubtedly the main target antigen in primary MN and is positive in 70% of pMN cases. The detection of its presence in the serum of patients demonstrates a sensitivity of 57%, especially in patients who have already undergone treatment; however, detecting its presence in renal biopsy material offers a more precise diagnostic method [19]. PLA2R detection methods in renal biopsy material using immunohistochemistry (IHC) and immunofluorescence (IF) for the differentiation of primary and secondary MN exhibit variable sensitivity and specificity. In 2014, Segarra-Medrano et al. compared the sensitivity of ELISA (74.5%), indirect IF (72.3%), and IHC (76.6%) [20], and Nicola M. Tomas and Laurence H. Beck et al. identified a glomerular protein (250 kDa in size) against which antibodies are formed in iMN, mainly in patients who tested negative for anti-PLA2R antibodies. Although this antigen is completely different from PLA2R, the two share some common biochemical characteristics. Spectrometry identified this antigen as thrombospondin type-1 domain-containing 7A (THSD7A) [21], while immunohistochemical analyses of renal biopsies revealed that THSD7A localizes on podocytes. Anti-THSD7A antibodies are detected in only 2% of adult patients with iMN, with a female predominance. The incidence of THSD7A-associated MN is 5.5% (7 of 117 PLA2R1-negative MN cases) according to immunoperoxidase staining, which was used to visualize the granular expression of THSD7A antigens [22]. The incidence was 6.1% (4 of 66) in a European cohort of PLA2R1-negative MN patients who had not received therapy [21]. Positive antibodies against PLA2R1 and THSD7A are found in 1% of patients with MN [22], and both types of antibodies are used as biomarkers for MN activity [23,24,25,26]. High levels of anti-PLA2R1 antibodies are considered a reliable prognostic factor that may alter therapeutic response and improve long-term treatment outcomes [18,27,28,29,30]. In 2017, Lu Pang et al. established that the serum anti-PLA2R antibody level is more closely correlated with disease activity and renal function than glomerular PLA2R deposition [31]. Patients who test positive for THSD7A require closer examination for neoplasia. In a study involving 1276 patients with MN, 8 women out of 40 patients with THSD7A-associated MN developed carcinoma within three months of diagnosis [32].

In 2019, Sanjeev Sethi et al. discovered new target antigens in MN, named Exostosin 1 (EXT1) and Exostosin 2 (EXT2) [33]. These antigens were initially identified in five patients who were PLA2R-negative using mass spectrometry and later confirmed by IHC. Subsequently, 209 PLA2R-negative patients were studied, including eight cases of PLA2R-negative membranous lupus nephritis, and 26 patients were positive for EXT1/EXT2. Clinical and biopsy data revealed interesting trends within EXT1/EXT2-associated MN patients. A total of 80.8% of these patients were women, with a mean age of 35.7 years, and 70.8% exhibited abnormalities in autoimmune laboratory tests, such as positive anti-nuclear, anti-double-stranded DNA, anti-SSA/SSB, or anti-ribonucleoprotein antibodies. A total of 35% had a clinical diagnosis of lupus and 12% had mixed connective tissue disease. The biopsy findings of 84.6% of patients indicated characteristics of autoimmune-related sMN, including positive staining for C1q and/or positive staining for IgA/IgM on IF, subendothelial and mesangial deposits, and tubuloreticular inclusions in endothelial cells as visualized with electron microscopy. The predominant IgG class was IgG1. It can be concluded that EXT1 and EXT2 are potential biomarkers or target antigens in secondary autoimmune MN.

In 2020, Tiffany N. Caza et al. discovered NCAM1, an immunoglobulin with a molecular weight of 120 kDa, using the method of Sethi et al. and improving upon it by incorporating protein G immunoprecipitation from frozen biopsies [34]. It was found that NCAM1 colocalizes with IgG in glomerular immune deposits. Unlike EXT1/EXT2, antibodies against recombinant NCAM1 were detected in human serum. NCAM1 was detected in 6.6% of patients with membranous lupus nephritis (MLN) and in 2% of patients with pMN. Of the NCAM1-positive patients with MLN, 40% had neurolupus, which is most likely related to the expression of NCAM1 in the central nervous system. NCAMI was not detected in normal kidney biopsies and non-NCAM1 MN cases. For this reason, NCAM1 is considered the second-best antigen/biomarker for MLN; however, an antigen was not identified in two-thirds of MLN patients.

NELL-1 is a 90 kDa neural epidermal growth factor-like 1 protein expressed in osteoblasts that promotes bone regeneration, which was discovered by Zhang X. et al. [35]. Sanjeev Sethi et al. studied 35 PLA2R-negative MN cases and detected NELL-1 in six cases using mass spectrometry [36]. They then tested these cases and 91 additional PLA2R-negative cases using immunohistochemistry (IHC) with NELL-1 staining, and 29 patients were found to be positive. It was observed that IgG and NELL-1 colocalizes on the glomerular basement membrane (GBM). Circulating antibodies against recombinant NELL-1 were detected in five patients and, in one patient, they were not detected after treatment with Rituximab, suggesting a correlation between antibody titer and disease activity.

Semaphorin 3B is a secreted 81 kDa protein belonging to a group of proteins that perform diverse functions during development; in adults, it acts through interaction with its receptors—plexin and neuropilin. In a collaborative study with pediatricians, Pierre Ronco and Hannaa Debiec reported positive staining for Semaphorin 3B in children with MN: 6/59 (10%) of the biopsies stained positive for Semaphorin 3B and also stained negative for PLA2R, THSD7A, NELL-1 and EXT1/2 [37]. When children with MLN (18/59) were not included, the true frequency of Semaphorin 3B was 14.9%, making Semaphorin 3B the top antigen in pediatric MN. In total, these authors identified 11 patients with Semaphorin 3B-associated MN, 8 of whom were children, and 5 were patients who had developed MN at or before 2 years of age. The mean age of the adult patients was 36.3 years, which was lower than the mean age of the pMN group. In all cases, granular staining of Semaphorin 3B was observed and was colocalized with IgG. Interestingly, in four cases—all pediatric—there were also granular deposits along the course of the tubular basement membrane.

In 2021, Moglie Le Quintrec et al. analyzed renal biopsies from five patients positive for antibodies against contactin 1 (CNTN1) and presenting with a chronic inflammatory demyelinating polyneuropathy [38]. Western blots revealed the expression of contactin 1 in normal renal glomeruli. Confocal microscopy analysis showed the presence and colocalization of contactin 1 and IgG4 on the glomerular basement membrane in these patients, while glomerular contactin 1 was absent in patients with anti-PLA2R1-associated MN or MLN and in healthy controls. However, the exact pathophysiology remains to be elucidated.

Protocadherin 7 (PCDH7) was identified in 2021 by Sethi et al. [39], who observed PCDH7-associated MN in the older age group, characterized by PCDH7 staining along the GBM. The IgG subclass against PCDH7 could be IgG1 or IgG4, and anti-PCDH7 antibodies were detected in serum. Interestingly, renal biopsy showed no or minimal complement deposition.

Serine protease HTRA1 (HTRA1) was discovered in 2021 by Laith Farah Al-Rabadi et al. As with PLA2R- and THSD7A-associated MN, anti-HTRA1 antibodies are predominantly of the IgG4 subclass, suggesting a primary etiology [40]. Analysis of sera collected during active disease versus remission revealed significantly higher titers of anti-HTRA1 antibodies during active disease. Screening of 118 “quadruple-negative” (PLA2R-, THSD7A-, NELL1-, EXT2-negative) patients from a large repository of MN biopsy specimens revealed a prevalence of 4.2%.

Transforming growth factor beta receptor 3 (TGFBR3) was identified in 2021 by Tiffany N Caza et al. in patients with MLN using mass spectrometry and laser capture microdissection, as well as via elution of immune complexes in the biopsy samples from these patients [41]. They discovered that TGFBR3 is enriched in glomeruli and coimmunoprecipitates with IgG in a subset of MLN biopsy specimens. Staining of consecutive MN cases without clinical evidence of SLE showed an absence of TGFBR3 expression (0 of 104), but a prevalence of 6% was identified in MLN (11 of 199 cases). TGFBR3 was observed to colocalize with IgG along the glomerular basement membrane in TGFBR3-associated MN cases but not in controls.

Netrin G1 (NTNG1), a membrane protein expressed in healthy podocytes, was identified as a novel target antigen in MN by Reinhard et al. in 2022 [42]. Immunohistochemistry confirmed granular NTNG1 positivity in subepithelial glomerular immune deposits. In prospective and retrospective cohorts of MN, three patients with NTNG1-associated MN were identified, who exhibited IgG4-dominant circulating NTNG1 autoantibodies, increased NTNG1 expression in the kidney, and IgG4 deposits. NTNG1 autoantibodies were not identified in 561 patients with positive PLA2R1 autoantibodies, 27 patients with positive THSD7A autoantibodies, or 77 patients with other glomerular diseases. In two patients available for follow-up for 2 and 4 years, NTNG1 autoantibodies persisted, along with proteinuria. The clinical role of NTNG1 autoantibodies remains to be defined.

Protocadherin FAT1 (FAT1) was identified in 2022 by Sanjeev Sethi et al. [43]. Using laser microdissection and tandem mass spectrometry (MS/MS), they identified this novel protein in nine patients with PLA2R-negative MN, all of whom developed MN after allogeneic hematopoietic stem cell transplantation. The authors then performed MS/MS in five patients already known to have allogeneic hematopoietic stem cell transplantation-associated MN and detected FAT1 in these patients. All 14 patients were negative for other known antigens on renal biopsy. Examination of renal biopsy specimens showed IgG along the GBM, with IgG4 being the dominant IgG subclass, and IHC confirmed granular FAT1 deposits along the GBM. Anti-FAT1 IgG was detected in the serum of those with FAT1-associated MN but not in those with PLA2R-associated MN.

Neuron-derived neurotrophic factor (NDNF) was identified in 2023, again by Sanjeev Sethi et al., via MS/MS in five patients with syphilis-induced MN [44].

Proprotein convertase subtilisin/kexin type 6 (PCSK6) was discovered in patients with long-term NSAID use through MS/MS in 2023 by Sanjeev Sethi et al. [45]. IHC/IF revealed granular staining of PCSK6 along the glomerular basement membrane, and confocal microscopy showed colocalization of IgG and PCSK6. Western blot analysis using frozen tissue eluates showed that IgG binds to PCSK6 in PCSK6-associated MN but not in PLA2R-positive MN.

In 2023, Sanjeev Sethi et al. proposed a new classification based on antigenic association [46], recommending the following terminology: “MN, target antigen-associated” for cases where a specific target antigen can be identified; “MN, undetermined antigen” for cases where no known antigen is identified; “MN, no antigen assessed” for cases where no associated antigens have been assessed; and “MN (list the antigens tested that are negative)” for cases where antigen assessment has been performed for a limited number of antigens and the results are negative. They recommend specifying which disease the detected antigen is associated with in parentheses.

## 3. Etiopathogenesis

Advances in etiopathogenesis have not been as successful as the discovery of antigens, despite extensive research efforts by numerous authors. It has been established that the target antigen in Heymann nephritis is megalin, a large (∼600 kDa) glycoprotein member of the LDL receptor family which consists of a large extracellular domain, a single transmembrane domain, and a short cytoplasmic domain [47,48]. The extracellular domain contains four putative functional regions, called ligand-binding domains, which consist of clusters of repeats characteristic of the LDL receptor gene family [47,48]. The four distinct ligand-binding domains contain epitopes to which antibodies are directed and, during the course of the disease, “epitope spreading” occurs [49]. Glycosylation of these epitopes plays a critical role in the pathogenicity of MN [50].

There are three pathways for complement activation. Activation of the classical pathway (CP) occurs through the binding of C1q and is usually observed in secondary forms of MN. However, pMN is characterized by the predominant deposition of IgG4, which has a very poor ability to bind to C1q. The amount of C1q detected in immune deposits in iMN is very low or not at all, suggesting that complement activation in iMN does not occur via the CP. In addition to the presence of IgG1 in immune deposits, which may activate the classical pathway in the early stages of the disease, some evidence suggests that alternative and/or lectin pathways may be more important in the development of the disease [51]. Hayashi et al. observed the deposition of mannose-binding lectin in glomeruli in iMN, and found that its intensity was correlated with the intensity of IgG4 staining [52]. Haddad et al. demonstrated that anti-PLA2R1 IgG4 autoantibodies could activate the lectin pathway and cause glycosylation-dependent damage to PLA2R1-expressing podocytes; these authors also identified the cellular pathways mediating this damage, and showed that patients with pMN exhibited abnormalities in IgG4 glycosylation that correlated with disease severity [53]. In 2024, it was proven that the activation of the lectin complement pathway plays a role in pathogenesis via IHC of MBL deposits in renal tissue, and this pathway prevails in the early stages of the disease. This MBL deposition was only observed in patients with IgG4 deposition. Activation of the lectin pathway was observed in pMN and iMN patients, but not in those with sMN [54].

In humans, PLA2R1 is expressed not only by podocytes but also by neutrophils, lung macrophages, airway epithelial cells, and submucosal epithelial cells. Wenbin et al. hypothesized that podocytes or other cells expressing PLA2R1 may be damaged and release extracellular vesicles, leading to the initiation of autoimmune activity and the development of MN [55]. Their hypothesis is that fine particulate matter with a diameter of 2.5 μm or less (PM2.5) induces the production of anti-PLA2R1 antibodies. The presence of PM2.5 in the airways and alveoli induces an inflammatory response involving neutrophils, alveolar macrophages, and epithelial cells. These authors postulate that these cells may express PLA2R1 which, due to oxidative stress and inflammation, undergoes conformational changes involving pathogenic epitopes, which further facilitates the formation of antibodies. Subsequently, PLA2R1 may be released into the extracellular space and bind to antigen-presenting cells, triggering a humoral immune response and the production of anti-PLA2R1 antibodies. This is followed by subepithelial deposition of immune complexes, triggered by anti-PLA2R1 antibodies produced outside the glomeruli. These antibodies cross the endothelial cells and the GBM, recognize and bind to PLA2R1 expressed on podocytes, and form immune complexes.

Xiaoying Hu et al., in their study on malignancy-associated MN, suggest that different types of tumors secrete tumor-associated antigens such as THSD7A, NELL-1, and PLA2R [56]. These antigens are ingested by antigen-presenting cells (APCs), processed into peptide segments, and presented to the APC surface. They then interact with helper T cells (Th cells) that specifically recognize B cells and promote the differentiation of B cells into mature plasma cells, which then secrete antibodies against THSD7A, NELL-1, and PLA2R. The resulting antigen–antibody immune complexes circulate and are deposited on the basement membrane of the glomerulus, causing MN.

Another hypothetical pathogenetic model of MN was published in 2025. In the study, the medical records of 102 patients with MN—57 men and 45 women—were reviewed retrospectively to establish correlations between comorbidities and MN. The patients were divided into three groups based on PLA2R positivity and clinical and paraclinical evaluations: pMN, iMN, and sMN. Comorbidities were grouped according to the organ from which they originated: diseases of the gall bladder, thyroid diseases, lung diseases, and liver diseases. The hypothesis is based on the fact that the PLA2R gene exhibits cell-type enrichment in different sites, and chronic inflammation in those sites (infectious, malignant, and autoimmune) leads to the formation of antibodies against PLA2R. Due to chronic inflammation, IgG4 is abnormally glycosylated. The immune system only recognizes the kidney as another site expressing PLA2R if conformational changes occur in PLA2R’s structure on the surface of podocytes. These changes are induced by the activation of the lectin pathway by abnormally glycosylated immunoglobulins, with additional triggers like NSAIDs, diabetes mellitus, and inflammation in two or more sites expressing PLA2R. Deposition of foreign antigens (tumor and immune complexes in SLE) in the kidney leads to complement activation, conformational changes in the structure of podocyte antigens, and the formation/sensitization of antibodies against them (not only against PLA2R but also other antigens on the podocyte surface). This explains the broad spectrum of antibodies discovered, as well as cases of double positivity for PLA2R and THSD7A and their appearance in pMN and sMN. This hypothesis is supported by the discovery of antibodies against PLA2R in the thyroid gland and gall bladder of patients with MN using IHC [57].

## 4. Connection Between the Antigens and Hypotheses Identified in the Literature

THSD7A is overexpressed in various types of malignant tumors, mainly in colorectal cancer, renal cancer, breast cancer, and prostate cancer [58]. The expression of PLA2R in tissues may be upregulated in chronic inflammatory or autoimmune diseases. Table S1 presents information on the cell-type enrichment of the identified antigens and their tissue protein expression; given that some of these antigens are also expressed in malignant diseases, antigens that are physiologically expressed in the corresponding organ or cell type in MN are highlighted using the same color as the associated tumor.

NELL1-positive MN is associated with lung carcinoma, thyroid carcinoma, colorectal carcinoma, and prostate carcinoma and is also linked to the use of skin-lightening creams [59]. Cell-type enrichment analysis of the NELL1 gene indicated its enrichment in hair cortex cells and eccrine sweat gland cells, both of which are also present in the skin. This distribution suggests a potential association with reported cases of MN following the use of skin-lightening creams.

SEMA3B is characteristic of the pediatric population with MN. Cell-type enrichment analysis of its gene indicated high expression in enteric glial cells. It is worth studying these children’s comorbidities and allergies in detail, as well as to look for the presence of coeliac disease, which can lead to the sensitization and recognition of SEMA3B as an antigen.

FAT1 antigens are present in patients with MN after HSCT. The presence of the FAT1 gene in stem and proliferating cells could possibly explain why MN appears after HSCT. Comparing MN without malignancy and MN with malignancy, its frequency rises from 1% to 92%, respectively [56]. HTRA1 genes are associated with cerebral small-vessel disease, age-related macular degeneration, tumors, arthritis, pre-eclampsia, and other microvascular and macrovascular diseases. The pathogenesis mainly arises due to an impaired vascular function [60]. The frequency of HTRA1 rises from 4% in MN without malignancy to 7% in MN with malignancy [56].

The information about these antigens’ gene cell-type enrichment and protein expression was collected from The Human Protein Atlas [61] and is organized and presented in Table S1 in the Supplementary Materials. The colored lines in some of the fields are used to highlight a connection or to emphasize a certain fact. The table is intended to illustrate additional sites of antigen expression when present and to indicate whether the patient has a chronic disease affecting an organ or tissue in which a specific antigen is expressed.

**Figure 2:**
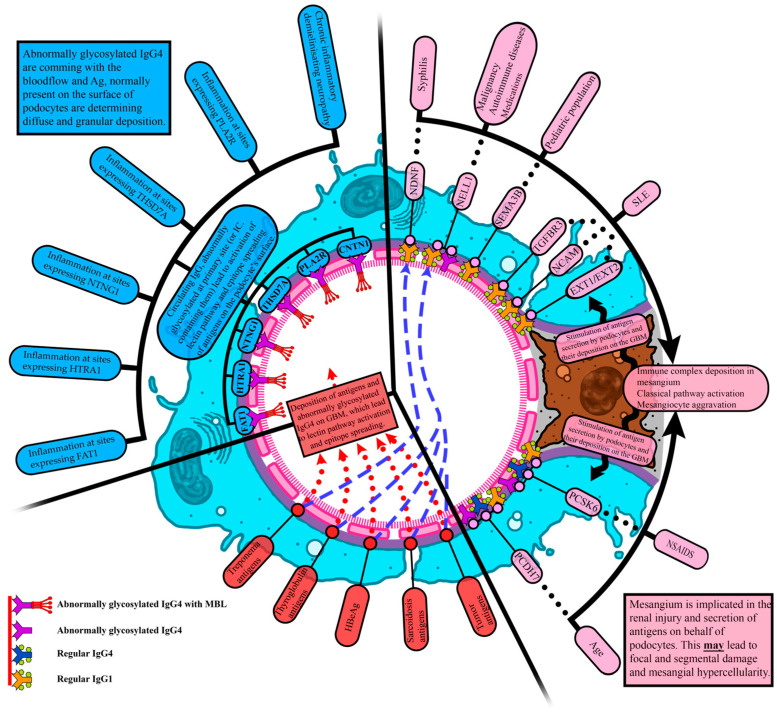
Pathogenetic model of membranous nephropathy. PLA2R—Phospholipase A2 Receptor; THSD7A—Thrombospondin Type-1 Domain-containing 7A; CNTN1—Contactin 1; HTRA1—HtrA serine peptidase 1; NTNG1—Netrin G1; FAT1—Protocadherin FAT1; SEMA3B—Semaphorin 3B; PCDH7—Protocadherin 7; NCAM1—Neural Cell Adhesion Molecule 1; NELL1—Neural Epidermal Growth Factor-Like 1; EXT1/EXT2—Exostosin-1/Exostosin-2; PCSK6—Proprotein Convertase Subtilisin/Kexin type 6; TGFBR3—Transforming Growth Factor Beta Receptor 3; NDNF—Neuron-derived Neurotrophic Factor.

Based on our findings and those of other researchers, we constructed a pathogenetic model of MN, which we believe will have practical application in future. This pathogenetic model is presented in Figure 2.

### 4.1. Explanation of the Proposed Pathogenetic Model

#### 4.1.1. Classical Pathway Damage

The damage is primarily induced by the activation of the classical pathway (CP) and secondarily by SLE, malignancies, autoimmune diseases, drugs, syphilis in the pediatric population and others. The activation of the CP in the mesangium stimulates podocytes to secrete NCAM, EXT1/EXT2, TGFBR3, NELL1, PCSK6, NDFN1, SEMA3B, and PCDH7. Mesangial and endocapillary hypercellularity and deposition of IgA, IgM, IgG, and C1q are frequently observed. The pattern of injury can be focal; the injury comes from the “outside” and podocytes react by secreting antigens, which are then deposited on the GBM subepithelially.

#### 4.1.2. Lectin Pathway Damage

The damage is induced by the activation of the lectin pathway (LP), arising from the abnormal glycosylation of IgG4. It is facilitated by NSAID intake, diabetes mellitus, and the production of IL-1 from podocytes. Activation of the LP leads to conformational changes in the structure of podocyte antigens, as well as the binding of IgG4 to their surface and epitope spreading. Inflammation of different origins (autoimmune, malignant, infectious) plays a key role at the primary site, where podocyte antigens are also expressed, leading to their recognition on a glomerular level. The injury comes from the “inside”, with blood flow facilitating a diffuse granular model of deposition. In this mode of injury, antigens are present on the podocyte surface or transmembrane or are GPI-linked. In the lectin pathway, podocyte injury due to external factors, such as NSAIDs and diabetes, plays an important role, leading to the deposition of PLA2R and other antigens on the surface of the GBM. These antigens are recognized by preformed antibodies, resulting in the activation of the lectin pathway. This speculation is supported by the fact that antibodies against PLA2R have been detected using IHC in tissues from the thyroid gland and gallbladder years before the appearance of MN [57]. In patients with diabetes, one of the most common non-diabetic injuries is focal segmental glomerulosclerosis (FSGS), followed by MN, as reported by Tamer Sakaci et al. [62] in their study of 159 patients with type 2 diabetes, among whom 64.8% had non-diabetic nephropathy.

With its increasing frequency, diabetes has become the leading cause of kidney injury because podocyte injury exposes nephrin, podocin, FAT1, and other antigens, which facilitates the formation of antibodies. This explains the high percentage of FSGS and MN in patients with diabetes. A great variety of agents, molecules, and genes have been implicated in podocyte injury at different levels, including actin reorganization and motility, podocyte polarity, apoptosis and mitochondrial dysfunction, autophagy, and actin-regulating proteins and enzymes [63].

#### 4.1.3. Intermittent Injury Pathway

In this mode of injury, the deposition of Treponema, sarcoidosis, tumor, and thyroglobulin antigens, as well as HBeAg, among others, on the GBM plays a key role. There are two different modes of action. In the first mode, antigen deposition on the GBM and abnormal IgG4 glycosylation, facilitated by diabetes mellitus and NSAIDs, can lead to LP activation, conformational changes in podocyte antigens, the formation of antibodies, and epitope spreading. Mesangial cells are implicated in the second mode of action, and preformed antibodies against antigens lead to inflammation and stimulate NDFN1 and NELL1 secretion on behalf of podocytes. We believe that PCSK6 and PCDH7 may also be involved in this intermittent pathway due to the presence of IgG1 and IgG4 against them.

### 4.2. Limitations of the Proposed Pathogenetic Model and Future Directions

The principal limitation of the proposed model is the difficulty of performing in vivo and in vitro experimental validation. Validation could be pursued using conditionally immortalized human podocyte cell lines, with individual antigens examined separately and exposed to antibodies derived from patients tested positive for the respective antigens. This approach would enable an assessment of the complement pathways involved in renal injury, as well as the identification of podocyte structures most vulnerable to complement-mediated damage. To achieve antigen expression levels comparable to those observed in human glomeruli in vivo, gene transfer techniques in differentiated cultured podocytes are likely required, as previously demonstrated by Haddad et al. [53].

In the proposed model, antigen-associated membranous nephropathies are conceptually stratified according to the pattern of IgG deposition. While physiologically glycosylated IgG4 does not activate the lectin pathway, aberrant IgG4 glycosylation—observed in autoimmune diseases, malignancies, and the natural aging process—may facilitate lectin pathway activation. We hypothesize that chronic inflammation in extrarenal organs promotes antibody formation, whereas subsequent renal injury, induced by factors such as non-steroidal anti-inflammatory drugs or diabetes mellitus, facilitates the recognition of the kidney as an additional site of antigen expression.

From this perspective, future experimental studies are warranted. Drawing on the paradigm of Heymann nephritis, one potential approach involves the induction of thyroiditis as an initial step, given that megalin is expressed in the thyroid, brain, and placenta. Subsequent experimental conditions could include diabetes mellitus, NSAID exposure, or their combination to model additional renal injury.

### 4.3. Current Management

A detailed clinical history remains a crucial component of the diagnostic workup, although its importance is increasingly underemphasized in routine practice. Structured questionnaires may therefore represent a useful tool, as comprehensive history taking can, in some cases, help to clarify the potential triggers or contributing factors to disease development. In our clinical experience, membranous nephropathy has been observed in patients following exposure to herbal weight-loss preparations, as well as in patients with gluten intolerance, in whom dietary indiscretions coincided with disease relapse and increased anti-PLA2R antibody levels. A sample questionnaire for patients with MN is provided in Table S2 in the Supplementary Materials.

The next evaluation steps include careful physical examination and appropriate instrumental investigations aimed at excluding secondary causes of MN. Laboratory assessment, in addition to standard tests, may include screening for syphilis, parasitic infections, tuberculosis, hepatitis, HIV infection, galactose intolerance, and coeliac disease, particularly in younger patients.

Immunological testing should be tailored to the assays available in each laboratory. Ideally, this panel should include antibodies against PLA2R, THSD7A, NELL1, SEMA3B, PCDH7, HTRA1, NTNG1, NCAM1, CNTN1, FAT1, NDNF, and PCSK6. Consideration may also be given to testing for anti-thyroglobulin and anti-thyroid peroxidase antibodies, as well as antibodies relevant to other autoimmune diseases. In selected cases, the additional testing of antibodies against podocin and nephrin may provide further information, as we have observed patients with membranous nephropathy who tested positive for podocin antibodies in addition to anti-PLA2R antibodies.

Renal biopsy remains the gold standard for diagnosis. Following confirmation of MN, IF for IgG4, and preferably combined IgG4/PLA2R staining, may be performed; alternatively, IHC for IgG4 and mannose-binding lectin (MBL) and, when available, anti-PLA2R staining, can be considered. Comprehensive testing for all known antigens in renal biopsy material is often not feasible. Consequently, in patients who are negative for circulating anti-PLA2R antibodies, a broader immunological panel may be informative, but only after histological confirmation of MN.

In the pediatric population, assessment of IgG subclasses is particularly important, as IgG1-dominant deposition has been described in TGFBR3-associated membranous nephropathy [46].

**Figure 3:**
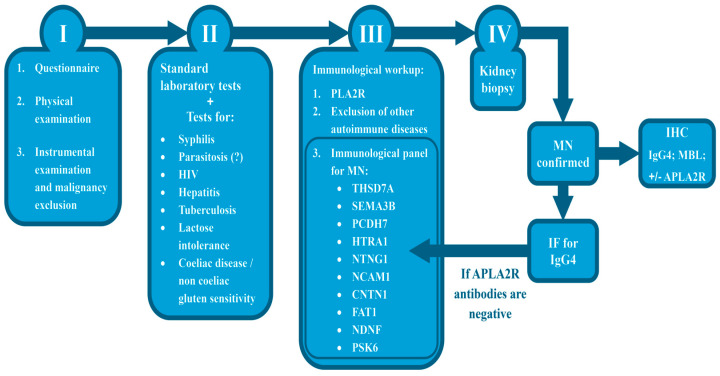
Algorithm for management of MN.

An algorithm for the management of MN, considering newly discovered antigens, is presented in Figure 3.

Given the limited availability of IHC staining for IgG4, MBL, or anti-PLA2R in some centers, and considering that MBL deposition may occur in early stages of disease, functional evaluation of the complement system represents an additional option. Complement screening assays assess the functional integrity of the classical, alternative, and lectin pathways by measuring their capacity to generate the terminal complement complex and may be most informative when performed prior to the initiation of pathogenetic treatment.

## 5. Discussion

The latest data on antigens and their expression in different types of MN have been discussed in this review. The feasibility of their use in routine daily practice can be determined only by the cost to the respective medical institution and the possibility of testing all these antigens and antibodies, in terms of the equipment and personnel required to process and interpret the results.

At present, each clinic/hospital, depending on its capabilities and focus (e.g., pediatric patients), determines its own panel of antibodies that can be tested. Regardless of the results, renal biopsies should be the gold standard, given the guidance they can provide regarding diagnoses of primary or secondary MN. There are guidelines to exclude SLE; the types of immune deposits and their localization in renal biopsies and the presence/absence of other clinical symptoms and involvement of other organs are also important to consider. Testing for oncological diseases in patients with MN should be part of routine screening, not only at the beginning but also at later stages of treatment, especially in elderly patients. Screening for infectious diseases such as syphilis, tuberculosis, and hepatitis should be considered part of routine clinical evaluation, not only because of their potential role in disease pathogenesis, but also because of the impact that pathogenetic treatment may have on their clinical course. Parasitosis and sarcoidosis should also not be forgotten. With our knowledge of MN and the discoveries made in recent years, there is scientific evidence for a probable mechanism that involves the kidneys. However, the importance of clinical work for patients with MN should not be overlooked. Medicine is evidence-based and instrumentally oriented, and it is important that, in our daily work, we are well informed and seek a balance between science and practice.

## 6. Conclusions

Sometimes we can both simplify and complicate the processes in the body in order to make them more logical and understandable. The truth is that the kidney is the main purifier in our body and “in vivo” and “in vitro” processes are distinct, as disease can be aggravated by other comorbidities, genetic predisposition, and the environment. It may be worthwhile to examine the complement pathway in serum and in kidney biopsies from patients with MN, as this may provide more clues regarding its pathogenesis and, just as importantly, help to differentiate primary from secondary MN.

## Data Availability

No new data were created or analyzed in this study. Data sharing is not applicable to this article.

## Supplementary Materials

The following supporting information can be downloaded at: https://www.mdpi.com/article/10.3390/ijms27052423/s1.

## Abbreviations

The following abbreviations are used in this manuscript:

MN	membranous nephropathy
PLA2R	phospholipase A2 receptor
iMN	idiopathic membranous nephropathy
sMN	secondary membranous nephropathy
SLE	systemic lupus erythematosus
AKI	acute kidney injury
eGFR	estimated glomerular filtration rate
ESRD	end-stage chronic kidney disease
NEP	neutral endopeptidase
APLA2R	anti-M-type phospholipase A2-receptor antibody
IHC	immunohistochemistry
IF	immunofluorescence
THSD7A	thrombospondin type-1 domain-containing 7A
EXT1	Exostosin 1
EXT2	Exostosin 2
NELL-1	neural epidermal growth factor-like 1 protein
Sema3B	Semaphorin 3B
NCAM1	neural cell adhesion molecule 1
MLN	membranous lupus nephritis
GBM	glomerular basement membrane
CNTN1	contactin 1
PCDH7	protocadherin 7
HTRA1	serine protease HTRA1
TGFBR3	transforming growth factor beta receptor 3
NTNG1	netrin G1
FAT1	protocadherin FAT1
MS/MS	microdissection and tandem mass spectrometry
NDNF	neuron-derived neurotrophic factor
PCSK6	proprotein convertase subtilisin/kexin type 6
PM2.5	fine particulate matter with a diameter of 2.5 micrometers or less
FSGS	focal segmental glomerulosclerosis
