# Supplementary figures and images for: Membranous Nephropathy: Antigenic Landscape and a Novel Pathogenetic Model
Source: Int J Mol Sci. 2026 Mar 6;27(5):2423. doi: 10.3390/ijms27052423 (PMC12985607; doi:10.3390/ijms27052423)

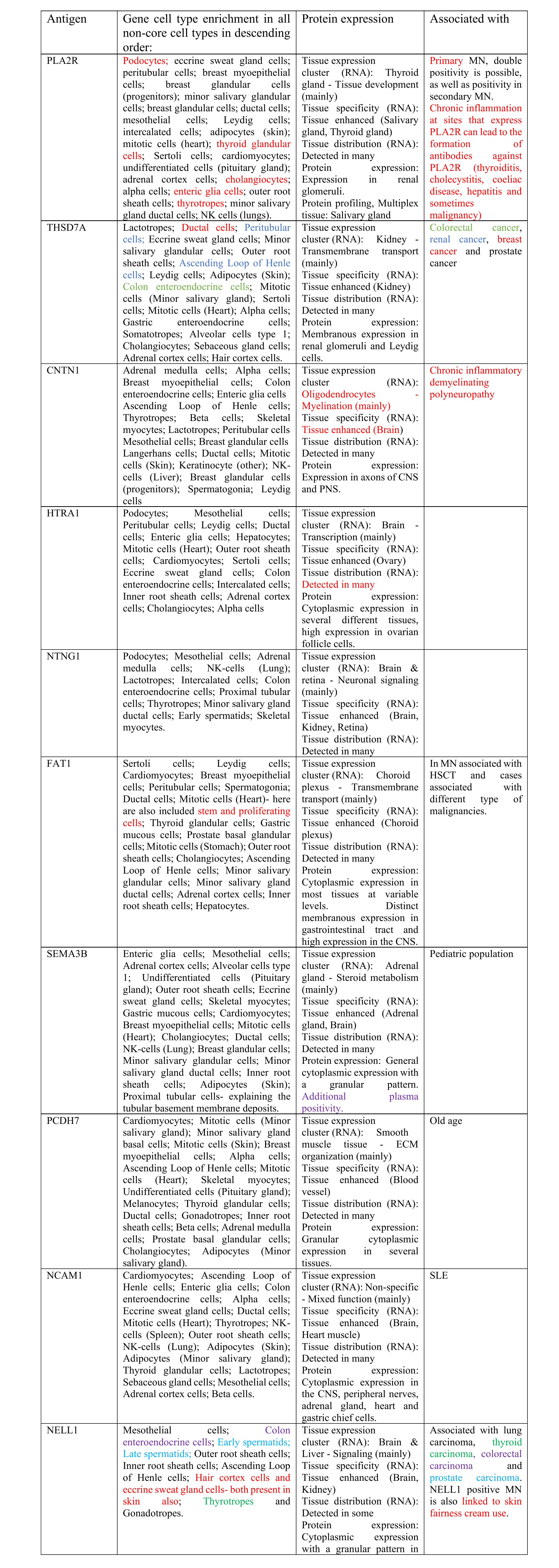

Supplement: Supplementary file 1 [file ijms-27-02423-s001.zip › Suplementary file/Table S1.png]

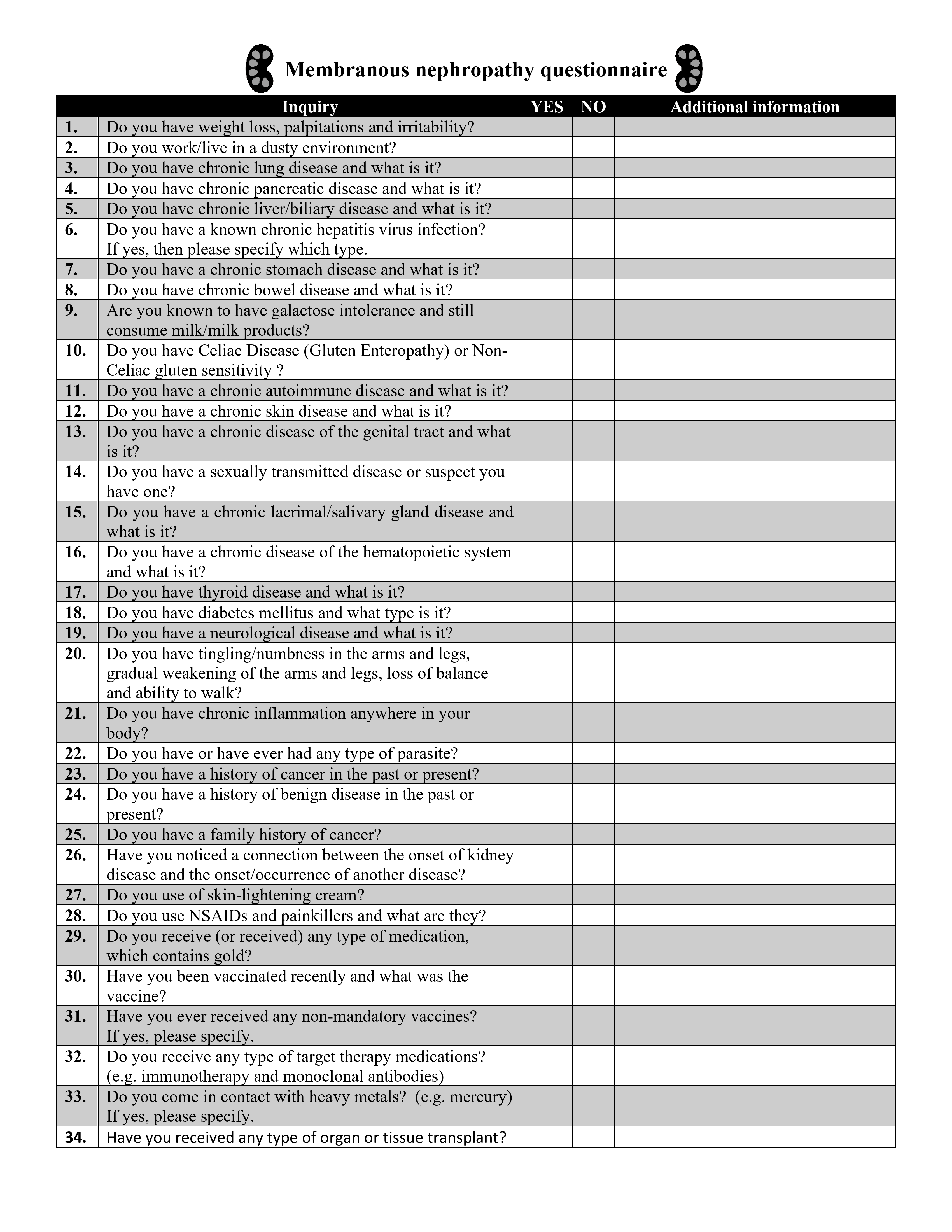

Supplement: Supplementary file 1 [file ijms-27-02423-s001.zip › Suplementary file/Table S2.png]
